# Sticky Platelet Syndrome in Patients with Uninduced Venous Thrombosis

**DOI:** 10.5152/tjh.2011.79

**Published:** 2013-03-05

**Authors:** Emre Tekgündüz, Muzaffer Demir, Alev Akyol Erikçi, Seval Akpınar, Erman Öztürk, Onur Kırkızlar

**Affiliations:** 1 Ankara Oncology Education and Research Hospital, Department of Hematology and Bone Marrow Transplantation Unit, Ankara, Turkey; 2 Trakya University, School of Medicine, Department of Internal Medicine, Division of Hematology, Edirne, Turkey; 3 Gülhane Military Medical School, Haydarpaşa Training Hospital, Department of Hematology, İstanbul, Turkey; 4 Göztepe Education and Research Hospital, Department of Hematology, İstanbul, Turkey

**Keywords:** Venous thrombosis, Blood platelet disorders, Platelet aggregation, Platelet function tests

## Abstract

**Objective:** Sticky platelet syndrome (SPS) is a common autosomal dominant inherited platelet disorder. SPS is characterized by platelet hyperreactivity and is associated with arterial and venous thrombosis. The aim of this study was to determine the role of SPS in patients with uninduced venous thrombosis.

**Material and Methods:** The study included 28 patients (15 male and 13 female) with uninduced venous thrombosis. SPS was defined according to Mammen’s aggregation method, which is described in detail elsewhere.

**Results:** According to the defined ranges for platelet hyperreactivity, 3 (50%) patients, 2 (33%), and 1 (17%) (n =6 [21%]) with a confirmed diagnosis were classified as type II, I, and III SPS, respectively. In 1 patient SPS was the only hereditary abnormality noted. The other 5 patients carried other inherited coagulation defects, in addition to SPS.

**Conclusion:** The present findings indicate that the prevalence of SPS was 21% in the patients with uninduced venous thrombosis. We therefore suggest that SPS should be considered in the differential diagnosis of such cases.

**Conflict of interest:**None declared.

## Abstract (tr)

**Amaç:** Yapışkan trombosit sendromu (YTS) trombosit hiperreaktivitesi ile karakterize sık rastlanan, otozomal baskın kalıtımlı, arteryel ve venöz tromboz ile ilişkili bir trombosit hastalığıdır. Bu çalışmanın temel amacı uyarılmamış venöz tromboz ile başvuran hastalarda YTS’nin rolünün belirlenmesidir.

**Gereç ve Yöntemler:** YTS’nin tanımlanmasında daha önce Mammen tarafından tanımlanmış olan agregasyon metodu kullanılmış ve çalışmaya uyarılmamış venöz trombozu olan 28 hasta (15 erkek ve 13 kadın) dahil edilmiştir.

**Bulgular:** Kendi normal değerlerimizi kullanarak trombosit hiperreaktivitesini değerlendirdiğimizde üç hasta (%50) tip II, iki hasta (%33) tip I ve bir hasta (%17) tip III YTS (toplam 6; %21) olarak sınıflandı. Bir hastada YTS saptanan tek kalıtsal bozukluktu. Beş hastada YTS’ye ek kalıtsal koagülasyon bozuklukları saptandı.

**Sonuç:** Sonuçlarımız uyarılmamış venöz tromboz olgularında YTS sıklığının %21 olduğunu ve YTS’nin bu hasta grubunun ayırıcı tanısında düşünülmesi gerektiğini göstermektedir.

## INTRODUCTION

Worldwide, venous thrombosis is a serious health problem associated with morbidity and mortality. In order to plan the long-term management and duration of anticoagulant therapy it is crucial to identify the underlying cause of thrombosis whenever possible. In about 50% of patients that present with venous thrombosis a hereditary or acquired coagulation defect, or a platelet disorder is present [1]. Sticky platelet syndrome (SPS) is a common hereditary platelet disorder with autosomal dominant inheritance. SPS is characterized by increased in vitro platelet hyperreactivity to adenosine diphosphate (ADP) and/or epinephrine (EPI) [2,3]. Patients with SPS present with arterial and/or venous thrombosis in various vascular beds. The diagnosis is based on platelet aggregation studies and in most patients it is easily treated with 100 mg/d aspirin.

Although the first patient with SPS was reported in 1983 [4], many physicians remain unfamiliar with this platelet defect and do not consider SPS when screening for thrombophilic risk factors in patients with unprovoked thrombosis. The present study we aimed to determine the role of SPS in patients that present with uninduced venous thrombosis.

## MATERIALS AND METHODS

**Study population**

The study was conducted between September 2006 and March 2009. During this period we consecutively enrolled all patients with unprovoked venous thrombosis that presented to our hematology outpatient clinic for etiological evaluation. The inclusion criteria were as follows: history of thrombosis ≥3 months prior to study entry; no other arterial/venous thromboembolic events during the 3 months prior to study entry; cessation of smoking ≥2 weeks before SPS evaluation; no use of any drug for ≥2 weeks, such as aspirin or non-steroidal anti-inflammatory drugs that interfere with platelet function; absence of any signs/symptoms indicative of an infection or inflammatory disease on the day of SPS evaluation; willing to participate.

The study was conducted in accordance with the Declaration of Helsinki and written informed consent was provided by all participants. The study protocol was approved by the Trakya University School of Medicine Ethics Review Board.

**Study protocol**

All patients with venous thrombosis that presented to our outpatient clinic for etiological work-up were evaluated via detailed history, physical examination, and appropriate laboratory testing for the presence of common acquired hypercoagulable states, including surgery, trauma, immobility, malignancy, pregnancy, hormone replacement therapy, catheter-induced thrombosis, nephrotic syndrome, myeloproliferative disorders.

Detailed imaging studies and invasive assessments for the detection of occult cancer were not performed. Consecutive patients without an identifiable cause for thrombosis were included in the study.

Aggregation studies were performed following overnight fasting and within 3 h of phlebotomy. Blood samples were drawn at 9.00 am after 15 min of resting. After the first 2 mL was discarded, blood was aspirated with a 19-gauge butterfly needle into a 20-mL syringe containing 2 mL of 3.2% sodium citrate solution. Platelet-rich (PRP) and -poor (PPP) plasma were obtained via centrifugation of anti-coagulated blood for 10 min at 100 g and 2000 g, respectively. PRP was mixed with PPP at appropriate volumes to obtain a standardized platelet count of 250,000 mm3; the stir bar speed was set at 1200 rpm.

SPS was defined according the method described by Mammen [2]. In brief, we pipetted 500 μL of PRP into a siliconized cuvette and placed another cuvette containing 500 μL of PPP into a blank chamber. We used 3 different ADP and EPI solutions as agonists. The final agonist concentrations in the testing chamber for ADP (Chrono-Par^®^) and EPI (Chrono-Par^®^) were 2.34 μM, 1.17 μM, and 0.58 μM, and 11 μM, 1.1 μM, and 0.55 μM, respectively. Aggregation reactions were recorded for 10 min after addition of agonists using a lumi-aggregometer (Chronolog^®^). The aggregation responses were recorded as aggregation percentage, with 100% being complete aggregation and 0% being no aggregation.

All participants were also screened for common inherited coagulation defects. Protein C and S, antithrombin activity, homocysteine levels, and the presence of antiphospholipid antibodies, activated protein C resistance (APCR), and prothrombin G20210A mutation were analyzed via standard methods, as described elsewhere [5]. Patients exhibiting APCR were assessed for factor V Leiden (FVL) mutation.

**Definition of SPS**

Standard criteria were used for the diagnosis and classification of SPS [2]. Normal aggregation responses in our laboratory were previously defined as part of another study (in review). Normal ranges of platelet aggregation were set as 10th-90th percentile values of healthy controls. History of thrombosis is a sine qua non criterion for SPS diagnosis. Apart from a history of thrombosis, hyperaggregable responses to ≥2 of the 6 agonist concentrations (ADP: 2.34, 1.17, and 0.58 μM; EPI: 11, 1.1, and 0.55 μM) confirmed the diagnosis of SPS. On the other hand, platelet hyperreactivity to only 1 concentration of ADP or EPI was accepted as suggestive of SPS. Suggestive cases were then reclassified as confirmed SPS if repeat testing was abnormal. Patients with abnormal responses to both reagents were classified as type I SPS, and patients with a hyperaggregable response to only ADP or EPI were classified as type II and III SPS, respectively.

## RESULTS

Patient demographic and clinical features are summarized in Table 1. The study included 15 male and 13 female patients with a median age of 39 years (range: 21-63 years) and 45 years (range: 29-60 years), respectively. In all, 5 (18%) patients were diagnosed as retinal vein thrombosis (RVT), 9 (28.5%) patients had pulmonary embolism (PE), 14 (50%) had deep vein thrombosis (DVT), and 1 (3.5%) patient had DVT complicated by PE. The most common genetic blood coagulation defect was FVL mutation. In total, 15 (54%) patients had FVL mutation. In 7 patients an inherited coagulation defect was not observed,whereas 2 patients had >1 hereditary coagulation defect contributing to thrombosis.

Normal aggregation response limits for SPS in our laboratory were determined during a previous study using blood samples obtained from 49 healthy controls, as mentioned above (Table 2). Six patients (21.5%) with a confirmed diagnosis of SPS and 6 (21.5%) with a suggestive diagnosis were identified accordingly. In total, 16 patients (57%) tested negative for SPS. Of the 6 patients with a confirmed diagnosis of SPS, 3 (50%), 2 (33%), and 1 (17%) were classified as type II, I, and III SPS, respectively. SPS was the sole hereditary thrombophilic abnormality in 1 of the patients with a confirmed diagnosis of SPS; the remaining 5 patients had other inherited coagulation defects, in addition to SPS.

## DISCUSSION

Venous thrombosis, due to its short- and long-term consequences, is a major health concern. As Virchow posited about 150 years ago, complex interaction of vascular stasis, hypercoagulability, and endothelial injury results in thrombosis at various locations. Based on history, physical examination, and modern laboratory techniques, a clinical condition, or a hereditary or acquired coagulation/platelet defect is the cause of venous thrombosis in nearly 80% of patients with venous thrombosis [2]. In general practice patients with the following conditions are candidates for evaluation of hereditary thrombophilia: unexplained venous thrombosis, VTE before age 50 years, family history of VTE, recurrent thrombosis, and thrombosis at unusual sites. Patients with the aforementioned conditions are frequently screened for the presence of APCR, prothrombin gene mutation, antiphospholipid antibodies, hyperhomocysteinemia, and protein C and S, and antithrombin deficiency; however, SPS is not routinely included in screening for thrombophilia. As SPS is a common disorder that can be effectively treated with 100 mg/d aspirin, it should be considered in the differential diagnosis of patients with unexplained venous or arterial thrombosis. 

As compared to hereditary coagulation defects, data on the role of SPS in arterial or venous thrombosis is scarce. Apart from venous thrombosis, SPS has been associated with various other clinical entities, including acute coronary syndrome with normal coronary angiography findings [6], transient ischemic attack [2], recurrent miscarriage syndrome [7], peripheral arterial microembolism [8], ischemic optic neuropathy [9,10] and post-transplant thromboembolic events [11,12]. The prevalence of SPS in patients with thrombosis varies with the location of involved vessels and study population. Among 599 consecutive patients with a new arterial or venous thrombotic attack, the prevalence of SPS was 20.5% [13]. Another study that included 159 patients with unexplained venous/ arterial thrombosis reported that the prevalence of SPS among those with retinal and deep vein thrombosis was 50% and 14%, respectively [2]. A study from Mexico that included 46 consecutive patients with unexplained thrombosis reported that 48% of the study cohort had SPS [14]. If we consider only a confirmed diagnosis of SPS, 40% (2/5) and 17% (4/23) of the present study’s patients that presented with RVT and DVT/PE had SPS, respectively; these results are in agreement with those of the studies mentioned above. As 6 of the present study’s patients with suggestive SPS refused to be retested, exclusion or confirmation of SPS in those patients was not possible, and they were considered suggestive cases.

Another important finding of the present study is that among the patients with a confirmed diagnosis of SPS (n=6), SPS was the sole thrombotic abnormality in only 1 patient (17% [3.5% of the entire study cohort]); in the other 5 patients SPS coexisted with other well known hereditary coagulation defects, which is similar to previous studies that reported that 83% [14] and 33% [15] of patients with SPS presented with additional congenital prothrombotic conditions.

We are well aware that the present patient cohort is too small for inferring definitive conclusions about the role of SPS in patients with unexplained venous thrombosis. The present study included only patients that presented with DVT, PE, and RVT; patients with thrombosis in other locations were not included. Furthermore, selection bias cannot be discounted, as we screened for SPS in patients that presented to our outpatient clinic. Venous thromboembolism is a multifactorial disorder. As the majority of our patients with confirmed diagnosis of SPS had other well known thrombophilic conditions, SPS in these patients should be interpreted as a contributing factor for development of thrombosis. Bearing these limitations in mind, the present study’s findings do provide some insight into the role of SPS in patients with unprovoked venous thrombosis in Turkey, and as such, physicians should be aware of this common inherited platelet defect that is presumed to lead to arterial and venous thrombosis. The present findings show that the prevalence of SPS in the patients that presented with uninduced thrombosis was high and that SPS should therefore be considered when screening for thrombophilia in such patients.

**Conflict of Interest Statement**

None of the authors has any conflicts of interest, including specific financial interests, relationships, and/or affiliations, relevant to the subject matter or materials included in this manuscript.

## Figures and Tables

**Table 1:**
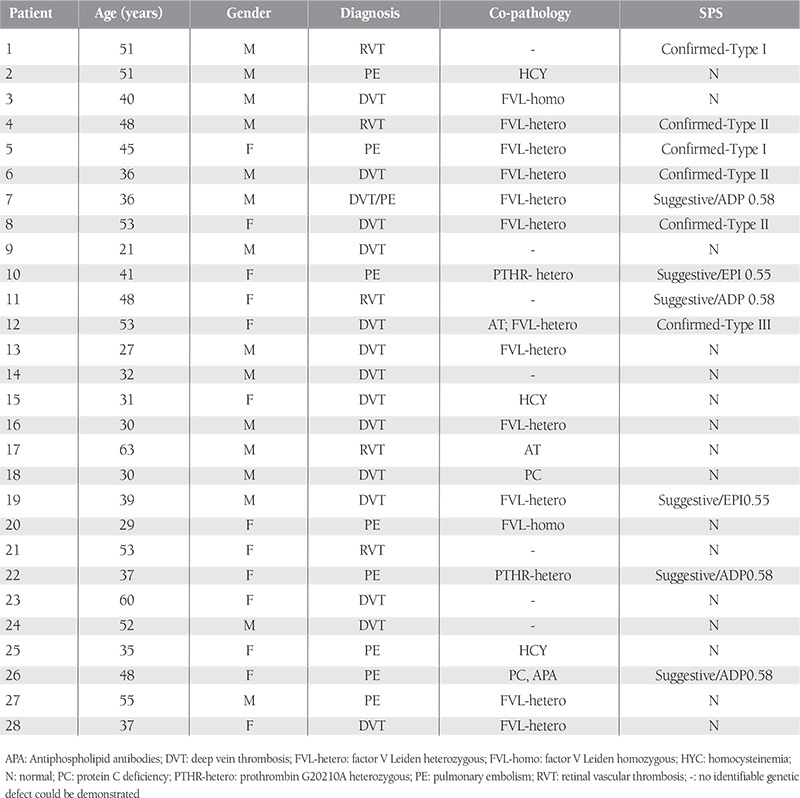
Study population demographic and clinical data.

**Table 2:**
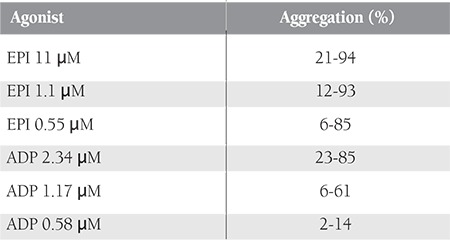
Normal aggregation ranges in our laboratory.
